# The Lessons of the Hospital Survey

**Published:** 1920-07-17

**Authors:** Arthur Stanley

**Affiliations:** 19 Berkeley Street, London, W. 1


### THE EDITOR'S LETTER-BOX.

**Correspondence on all subjects is invited, but we cannot in any way be responsible for the opinions expressed by our correspondents, who must give their name and address as a guarantee of good faith, but not necessarily for publication. Correspondents are reminded that brevity of style and conciseness of statement greatly facilitate early insertion.**

### The Lessons of the Hospital Survey.

*To the Editor of* THE HOSPITAL.

Sir,—Sir Napier Burnett has now finished his preliminary survey of the finances of the Provincial Hospitals in England, Wales, Scotland, and Ireland, and attached herewith is his statement. For the first time it is possible to form some idea of the needs not of the individual hospitals only but of the whole hospital service: In other words, we can really see what is being done for the sick of the United Kingdom and Ireland, what still remains to be done, how much money is now being found from voluntary sources, and how much more remains to be found.

If the voluntary system is to continue five things are necessary:

1. Payment of war debts preferably by existing voluntary organisations, or, failing that, by a grant from the Exchequer.

2. The extension of the almoners' system, under which a contribution towards their maintenance is requested from those patients who can afford to make it.

3. The organisation of systematic collections from work-people and employers of labour.

4. The establishment of a competent authority to examine hospital accounts and consolidate appeals.

5. Closer co-operation with the Poor-law infirmaries, in which there is a large number of vacant beds that might be utilised to relieve the pressure on existing voluntary hospitals.

One fact is quite clear, and that is that the voluntary system is not bankrupt. True, there are necessarily large accumulations of repairs and renewals to be faced, but if, as suggested in the conclusions at the end of Sir Napier Burnett's letter, the legacy of debt left by five years of war can be removed from the shoulders of the hospitals I feel confident that the subscriptions and donations of the public, which were sufficient to maintain the hospitals in pre-war days, will expand to meet the growing needs and the great increase in the price of all commodities.

The immediate and urgent need is one million pounds to discharge the legacy of the war, and to give the nation's hospitals a fresh and clean start in their race with sickness and disease.—Yours faithfully,

Arthur Stanley.

19 Berkeley Street, London, W. 1,

July 1920.

